# Supplementary material for: Biallelic variants in MAD2L1BP (p31comet) cause female infertility characterized by oocyte maturation arrest
Source: eLife. 2023 Jun 19;12:e85649. doi: 10.7554/eLife.85649 (PMC10319434; doi:10.7554/eLife.85649)
Supplement: Table 2—source data 1. [file elife-85649-table2-data1.zip › Table 2 -source data 1.docx]

Homozygous and compound heterozygous variants identified by WES that survived filtering in Patient **F1: II-1**

| **Genomic SNV** | **Gene** | **Change** | **phastCons score** | **ExAC allele frequency** | **ExAC homozygotes frequency** | **gnomAD allele frequency** | **gnomAD homozygotes frequency** | **SIFT** | **Polyphen** | **Mutation**  **Taster** | **In homozygous region** | **OMIM** |
| --- | --- | --- | --- | --- | --- | --- | --- | --- | --- | --- | --- | --- |
| Chr3: 183754539 G>A | HTR3D | c.515G>A:p.R172Q | 0.981^a^ | 3/23520 | 0/23520 | 6/159876 | 0/159876 | 0.02(D)^b^ | 0.005 (N)^b^ | 1(N)^c^ | 0.56 Mb | - |
| Chr1: 247978306 G>T | OR14A16 | c.726C>A:p.H242Q | 0.190 | 15/121206 | 0/121206 | 23/282554 | 0/282554 | 0.01(D)^c^ | 0.998 (D) | 1(N) | 0.44 Mb | - |
| Chr17: 39296642 G>A | KRTAP4-6 | c.98C>T:p.T33I | 0.585 | 4/118336 | 0/118336 | 8/249342 | 0/249342 | 0.04(D) | - | 0.989(N) | 0.30 Mb | - |
| Chr19: 51628494 G>A | SIGLEC9 | c.263G>A:p.R88Q | 0.002 | Not found | Not found | 2/282782 | 0/282782 | 0.83(N) | 0.003 (N) | 1(N) | 1.19 Mb | - |
| Chr9: 139995555 C>T | MAN1B1 | c.1015C>T:p.L339F | 0.990 | Not found | Not found | Not found | Not found | - | 0.999 (D) | 1(D) | 0.05 Mb | Mental retardation, autosomal recessive (614202) |
| Chr6: 43608202 C>T | MAD2L1BP | c.853C>T:p.R285* | 0.063 | 2/117786 | 0/117786 | 3/248598 | 0/248598 | - | - | 1(D) | 3.82 Mb | - |
| Chr16: 2347798 G>C | ABCA3 | c.2021C>G:p.S674C | 1.000 | Not found | Not found | Not found | Not found | 0.03 (D) | 0.898 (P) | 1(D) | - | Neonatal and pediatric interstitial lung disease, autosomal recessive, (610921) |
| Chr16: 2347793 C>T | ABCA3 | c.2026G>A:p.G676S | 1.000 | 1/120546 | 0/120546 | 13/282418 | 0/282418 | 0.51 (N) | 0.745 (P) | 1(D) | - |  |
| Chr4: 7435334 C>A | PSAPL1 | c.1273G>T:p.G425C | 0.420 | 2/104104 | 0/104104 | 4/244564 | 0/244564 | 0.00 (D) | 0.829 (P) | 0.998(N) | - |  |
| Chr4: 7435697 G>C | PSAPL1 | c.910C>G:p.L304V | 0.086 | Not found | Not found | 2/248922 | 0/248922 | 0.63 (N) | 0.446 (N) | 1(N) | - |  |
| Chr16: 78062033 G>T | CLEC3A | c.172G>T:p.E58* | 1.000 | 1/120624 | 0/120624 | 3/280050 | 0/280050 | 0.03 (D) | - | 1(D) | - |  |
| Chr16: 78062096 C>A | CLEC3A | c.226+9C>A^d^ | 0.000 | 6/118912 | 0/118912 | 20/248102 | 0/248102 | - | - | - | - |  |

**a**. phastCons score reveals the conversation of the site. The data were based on 100 vertebrates conservation by PhastCons (phastCons100way) in UCSC. The scores were higher; the possibilities of being deleterious for the variants will be higher. **b**. Scores were lower in SIFT or higher in either Polyphen, the possibilities of being deleterious (D) for the mutations will be higher. **c**. Scores were higher in MutationTaster^4^, the possibilities of being neutral (N) for the mutations will be higher. **d.** NNSplice reveals no change of the donor site score for the variant.

Homozygous and compound heterozygous variants identified by WES that survived filtering in Patient **F2: II-1**

| **Genomic SNV** | **Gene** | **Change** | **phastCons score** | **gnomAD allele frequency** | **gnomAD homozygotes frequency** | **SIFT** | **Polyphen** | **Mutation**  **Taster** | **Homo-/Hetero-** | **OMIM** |
| --- | --- | --- | --- | --- | --- | --- | --- | --- | --- | --- |
| Chr1: 248458874 delC | OR2T12 | c.6delG;p.E2Efs*^a^ | 0.000 | 2/211788 | 0/211788 | - | - | - | Homo- | - |
| Chr11:209897 insA | RIC8A | c.623_624insA; p.P208Pfs9* | 0.008 | Not found | Not found | - | - | - | Homo- | - |
| Chr21:45970774 G>A | KRTAP10-2 | c.568C>T;p.P190S | 0.102 | Not found | Not found | 0.054(T) | 0.053(B) | 1(N) | Homo- | - |
| Chr11:76895792 T>G | MYO7A | c.3535T>G; p.*1179G | 0.000 | Not found | Not found | - | - | 1(N) | Homo- | Usher syndrome, type 1B, 276900, Autosomal recessive |
| Chr19: 501743 T>C | MADCAM1 | c.742T>C; p.S248P | 0.000 | Not found | Not found | 0.312(T) | 0.0(B) | 0.0(B) | Homo- | **-** |
| Chr20:62196096 C>T | HELZ2 | c.4079G>A; p.R1360Q | 0.055 | 140/277396 | 0/277396 | 0.099(T) | 0.385(B) | 0.999909(N) | Hetero- |  |
| Chr20:62194368 C>T | HELZ2 | c.5807G>A; p.R1936H | 0.000 | 5/255676 | 0/255676 | 0.256(T) | 0.004(B) | 1(N) | Hetero- |  |
| Chr20:62200284 G>A | HELZ2 | c.1157C>T; p.A386V | 0.969 | 8/271120 | 0/271120 | 0.025(D) | 0.202(B) | 1(N) | Hetero- |  |
| Chr5:135229738 C>T | IL9 | c.286G>A; p.V96I | 0.000 | 188/281554 | 0/281554 | 0.097(T) | 0.221(B) | 1(N) | Hetero- | IL-9 is a candidate gene for asthma. |
| Chr5:135229769 G>C | IL9 | c.255C>G; p.Y85* | 0.000 | 190/282292 | 0/282292 | - | - | 1(A) | Hetero- |  |
| Chr19:48637292 C>A | LIG1 | c.1552G>T; p.V518L | 0.984 | 8/165242 | 0/165242 | 0.083(T) | 0.144(B) | 0.999965(D) | Hetero- | DNA ligase I deficiency, autosomal recessive. |
| Chr19:48664717 G>A | LIG1 | c.155C>T; p.P52L | 0.992 | 20/251384 | 0/251384 | 0.0(D) | 0.998(D) | 0.99995(D) | Hetero- |  |
| Chr3:52555444 T>A | STAB1 | c.5976T>A; p.S1992R | 0.000 | 36/282120 | 0/282120 | 0.045(D) | 0.927(D) | 0.997905(N) | Hetero- |  |
| Chr3:52555443 G>C | STAB1 | c.5975G>C; p.S1992T | 0.118 | 36/282164 | 0/282164 | 1.0(T) | 0.054(B) | 0.999992(N) | Hetero- |  |
| ***Chr6: 43607867delT*** | ***MAD2L1BP*** | ***c.518delT; p.F173Sfs4**** | ***1.000*** | ***Not found*** | ***Not found*** | ***-*** | ***-*** | ***-*** | Hetero- |  |
| ***Chr6: 43600837G>A^b^*** | ***MAD2L1BP*** | ***c.21-94G>A^c^*** | ***0.000*** | ***111/31250*** | ***2/31250^d^*** | ***-*** | ***-*** | ***-*** | Hetero- |  |

1. If the first downstream alternative AUG initiation site in the open reading frame (ORF) of mRNA was used, the variant chr1: 248458874 delC would be benign and the produced protein would be functional with the deletion of the first two amino acids. In addition, chr1: 248458875delT (p.E2Gfs2*) has a high gnomAD homozygotes frequency 5156/226096, therefore the variant chr1: 248458874 delC of OR2T12 could be tolerated. **b.** The heterozygous variants in *MAD2L1BP* was initially filtered out. **c.** The variant c.21-94G>A is predicted to introduce an alternative splice acceptor by NNsplice (the increase of the acceptor site score from 0 up to 0.13) and by ASSP(the increase of the acceptor site score from <2.2 up to 7.078). **d.** Two homozygotes include one female and one male, and both are of Latino/Admixed Americian origin.

Homozygous and compound heterozygous variants identified by WES that survived filtering in Patient **F3: II-1**

| **Genomic SNV** | **Gene** | **Change** | **phastCons score** | **gnomAD allele frequency** | **gnomAD homozygotes frequency** | **SIFT** | **Polyphen** | **Mutation**  **Taster** | **Homo-/Hetero-** | **OMIM** |
| --- | --- | --- | --- | --- | --- | --- | --- | --- | --- | --- |
| Chr5:112824027_112824028 insGCTGCTGCTGCC | MCC | c.84_85insGGCAGCAGCAGC; p.S29_D30insGSSS | 0.992 | Not found | Not found | - | - | - | Homo- | Colorectal cancer, somatic, 114500 (3) |
| Chr1:55631552 A>T | USP24 | c.927+6T>A | 1.000 | 134/269132 | 2/269132 | - | - | - | Homo- | Ubiquitin-specific protease 24, 610569 |
| Chr6:43607890 C>T | MAD2L1BP | c.C541T; p.R181* | 0.992 | 2/250850 | 0/250850 | - | - | 1(D) | Homo- | - |
| Chr1:228556122 G>A | OBSCN | c.22638+5G>A | 0.000 | 29/245048 | 1/245048 | - | - | - | Hetero- | - |
| Chr1:228558958 C>T | OBSCN | c.C23350T; p.R7784W | 0.000 | 126/230436 | 0/230436 | 0.005(D) | 0.798(P) | 0.9999(N) | Hetero- | - |
| Chr5:160033918 G>T | ATP10B | c.C3014A; p.A1005D | 0.102 | 16/280850 | 0/280850 | 0.064(T) | 0.457(P) | 0.980394(N) | Hetero- | - |
| Chr5:160063269 C>T | ATP10B | G1048A; p.V350M | 0.976 | 2/280128 | 0/280128 | 0.002(D) | 0.999(D) | 1(D) | Hetero- | - |
| Chr9:135946948 T>G | CEL | c.T2068G; p.S690A | 0.000 | 20/91304 | 0/91304 | 0.0(D) | 0.841(P) | 1(N) | Hetero- | Maturity-onset diabetes of the young, type VIII, 609812 (3), Autosomal dominant |
| Chr9:135946954 G>C | CEL | c.G2074C; p.A692P | 0.000 | 1/9734 | 0/9734 | 0.0(D) | 0.999(D) | 1(N) | Hetero- |  |
| Chr16:1573631 G>A | IFT140 | c.C3341T; p.A1114V | 1.000 | Not found | Not found | 0.03(D) | 0.999(D) | 1(D) | Hetero- | Short-rib thoracic dysplasia 9 with or without polydactyly, 266920 (3), Autosomal recessive |
| Chr16:1642478 G>T | IFT140 | c.C481A; p.P161T | 0.669 | 13/282578 | 0/282578 | - | 0.004(B) | 0.999821(D) | Hetero- |  |
