# Supplementary material for: Biallelic variants in MAD2L1BP (p31comet) cause female infertility characterized by oocyte maturation arrest
Source: eLife. 2023 Jun 19;12:e85649. doi: 10.7554/eLife.85649 (PMC10319434; doi:10.7554/eLife.85649)

**
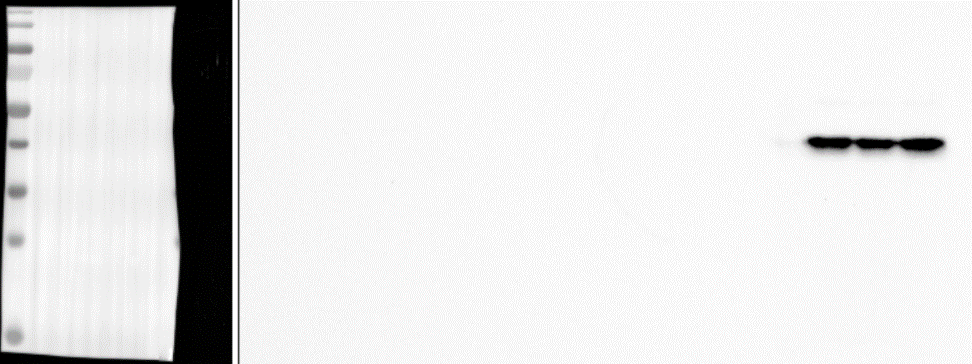

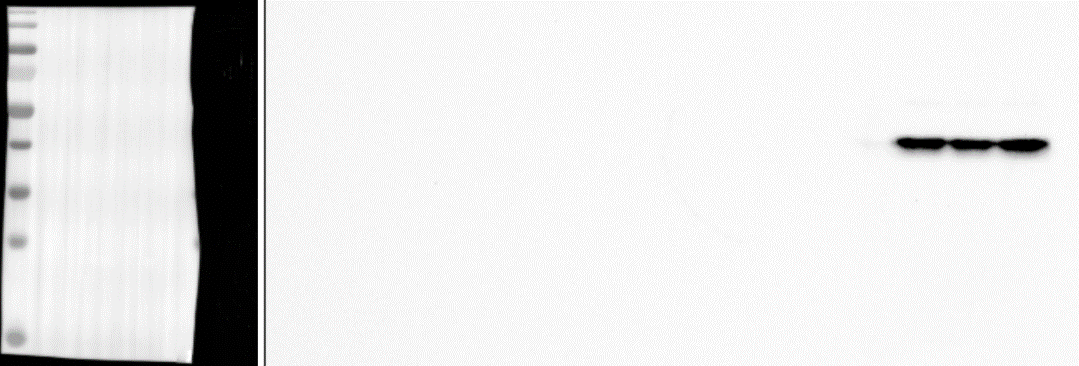

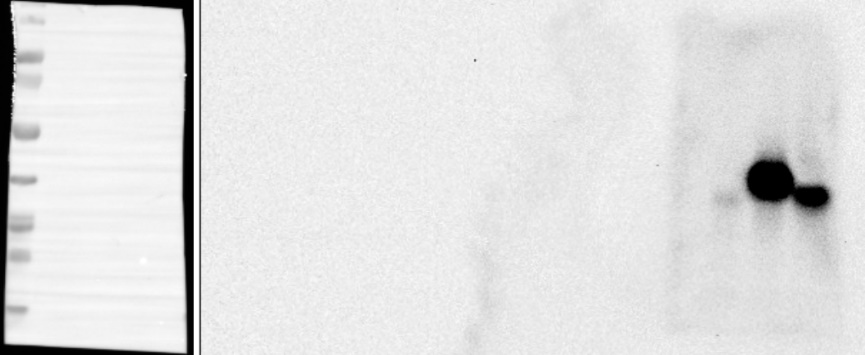

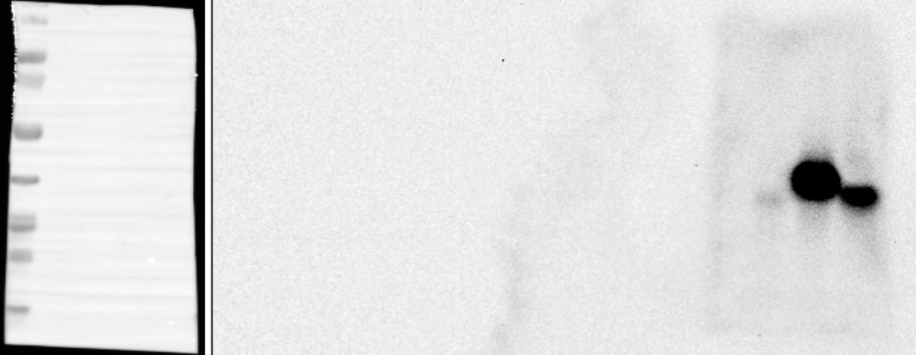

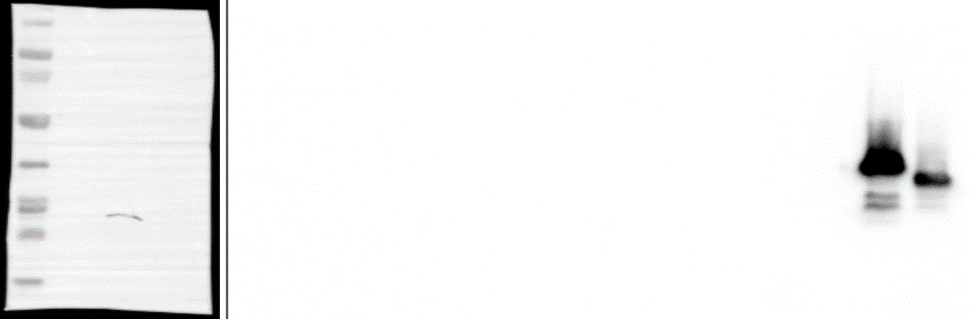

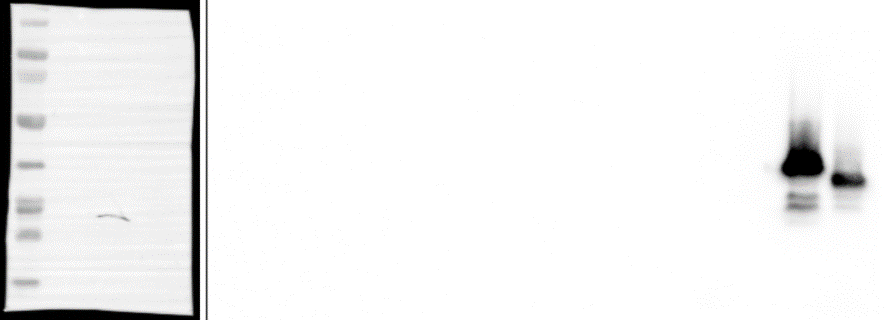

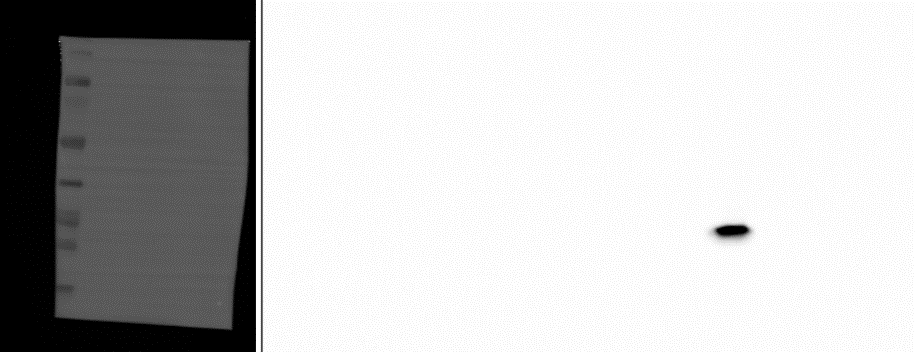

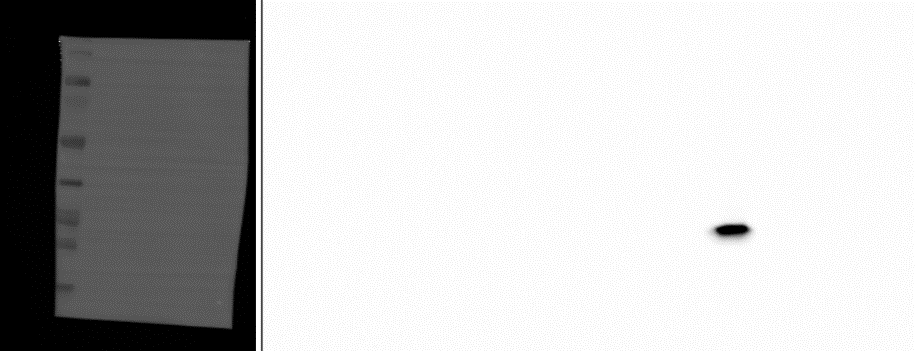
**

**Anti-β-actin**

Flag-MAD2L1BP MUT

**Anti-MAD2L1BP**

**Anti-Flag**

**Anti-Myc**

Myc-MAD2

Flag-MAD2L1BP WT

Flag-MAD2L1BP MUT

Flag-MAD2L1BP WT

Myc-MAD2

Flag-MAD2L1BP MUT

Flag-MAD2L1BP WT

Myc-MAD2

Flag-MAD2L1BP MUT

Flag-MAD2L1BP WT

Myc-MAD2

35KD

42KD

55KD

70KD

100KD

130KD

25KD

15KD

42KD

35KD

25KD

15KD

55KD

70KD

100KD

130KD

Marker

Marker

Marker

15KD

25KD

35KD

42KD

55KD

70KD

100KD

130KD

Marker

15KD

25KD

35KD

42KD

55KD

70KD

100KD

130KD


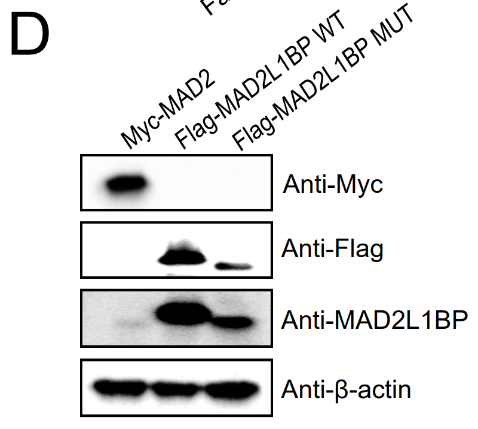

Supplement: Figure 2—source data 1. [file elife-85649-fig2-data1.zip › Figure 2-source data 1.docx]
