# Supplementary material for: Biallelic variants in MAD2L1BP (p31comet) cause female infertility characterized by oocyte maturation arrest
Source: eLife. 2023 Jun 19;12:e85649. doi: 10.7554/eLife.85649 (PMC10319434; doi:10.7554/eLife.85649)
Supplement: Figure 2—source data 2. [file elife-85649-fig2-data2.zip › Figure 2-source data 2.docx]

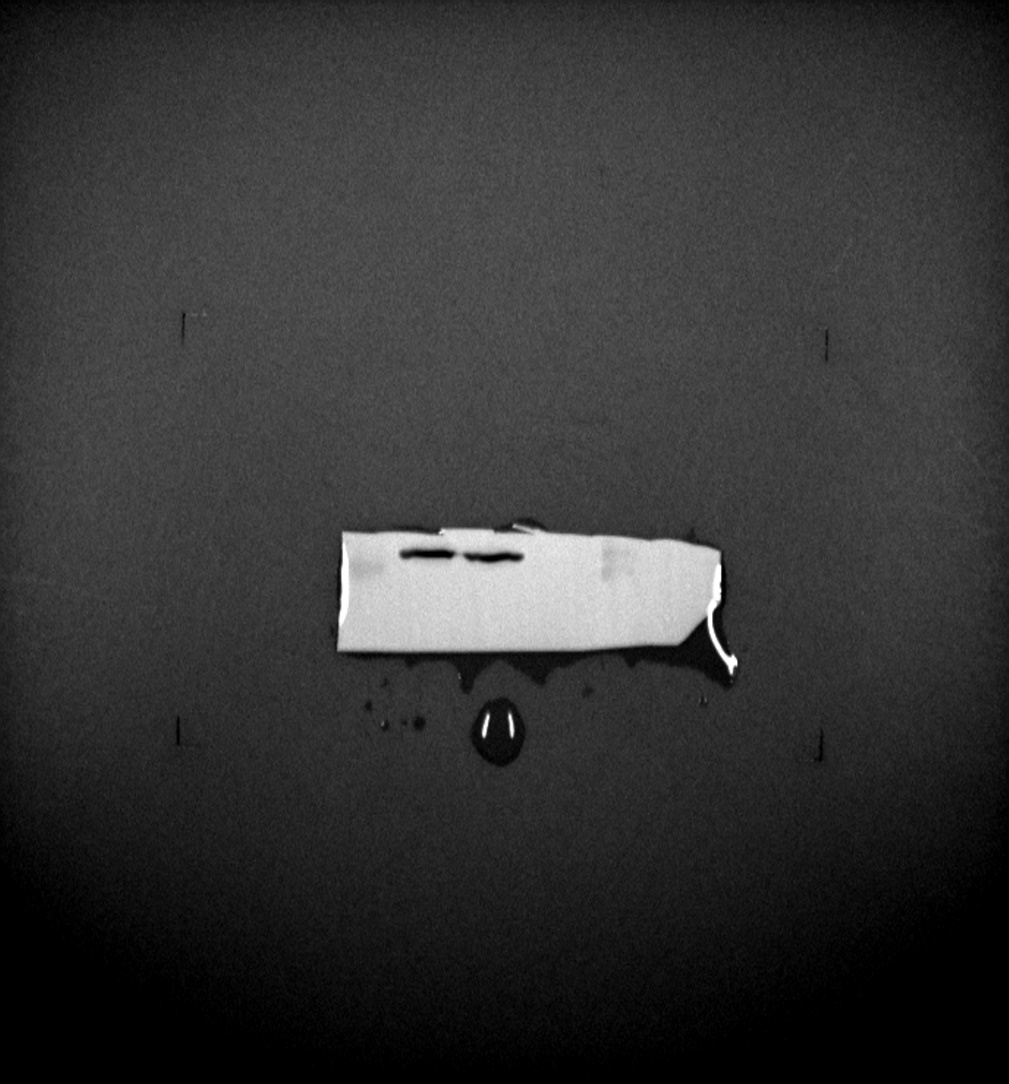

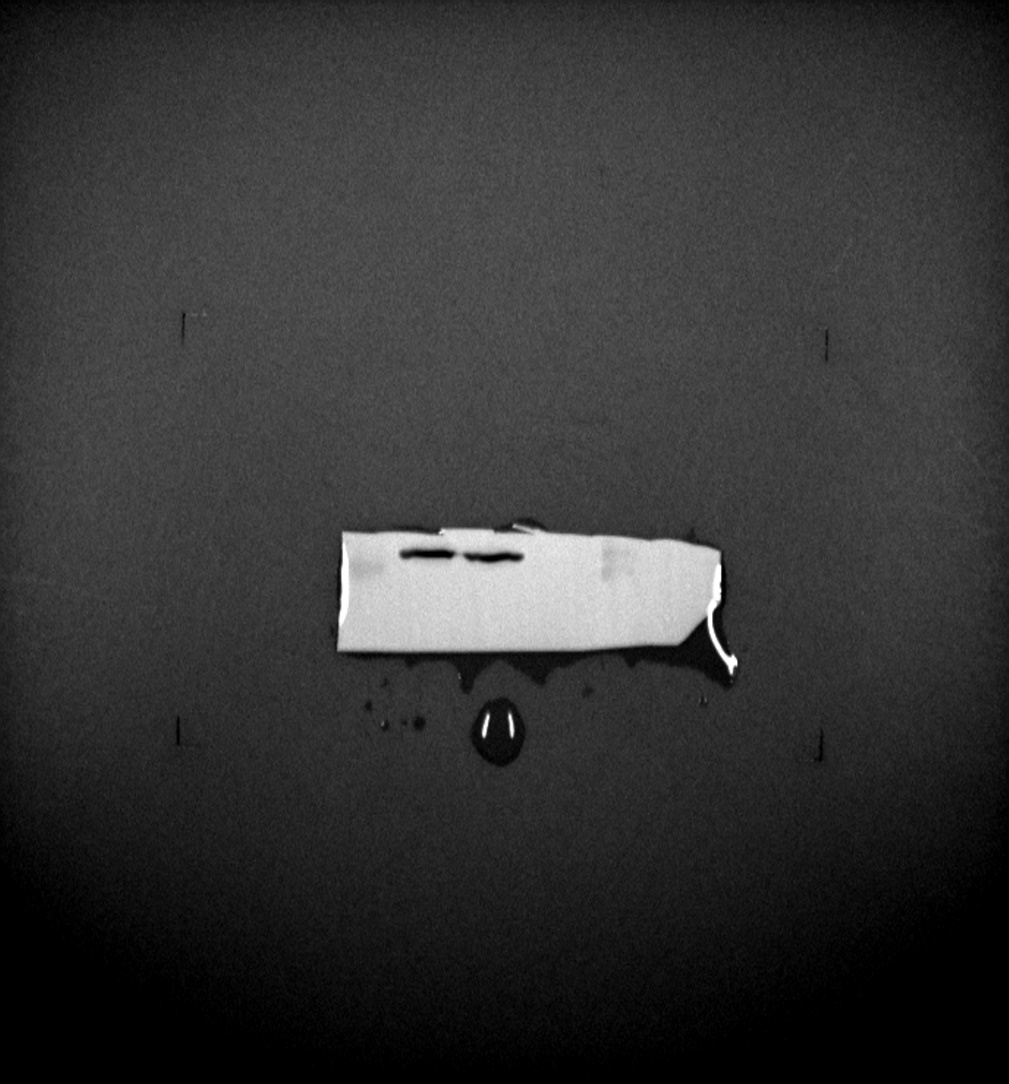

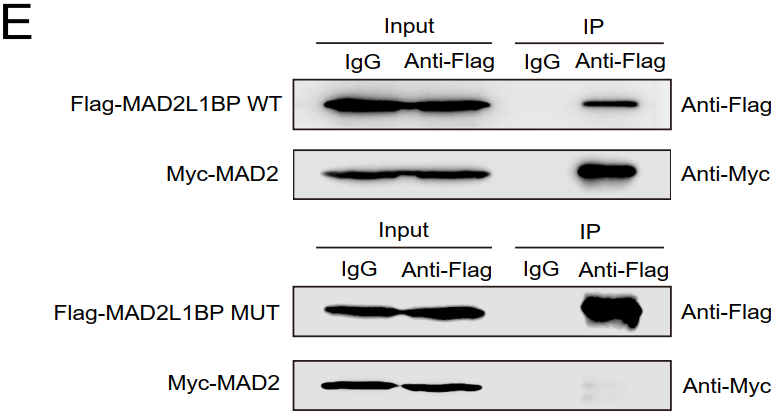

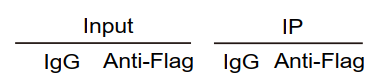

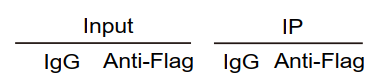

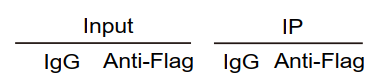

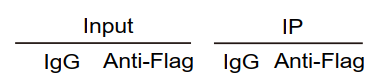

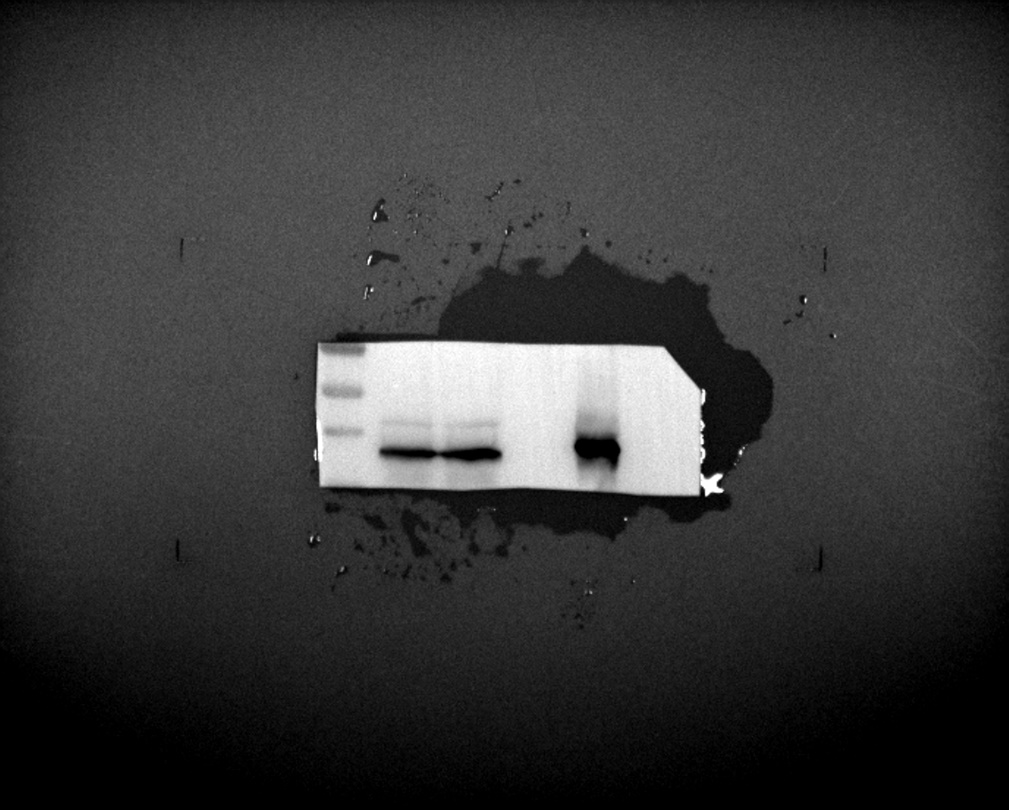

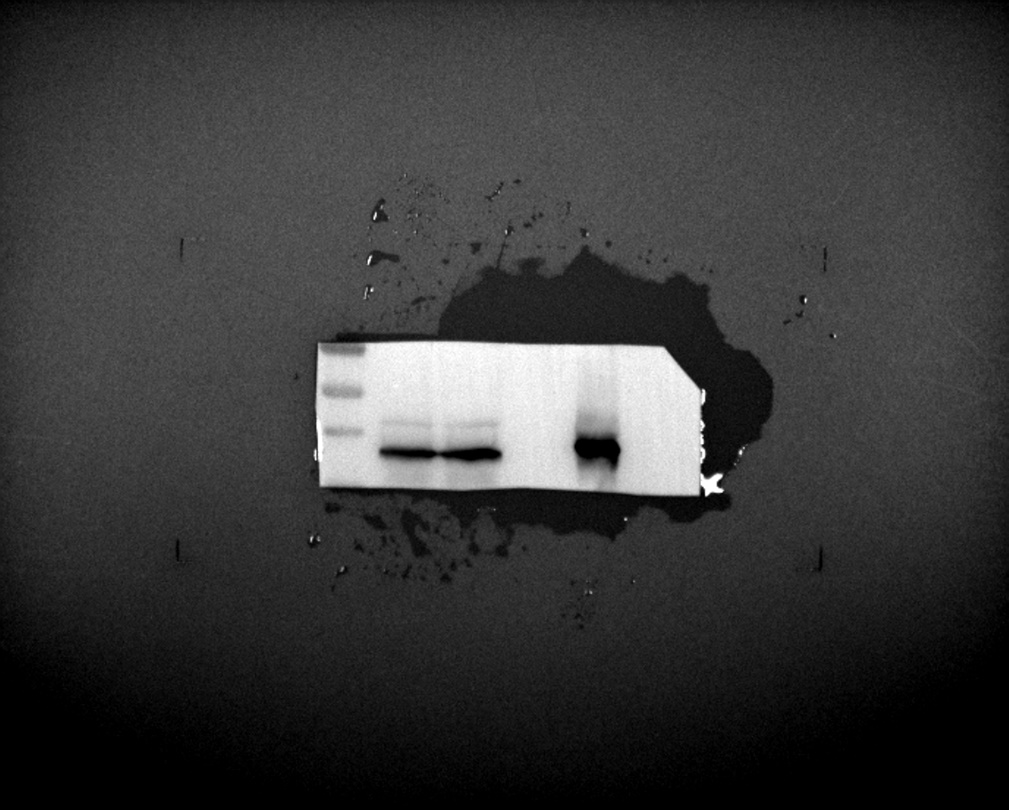

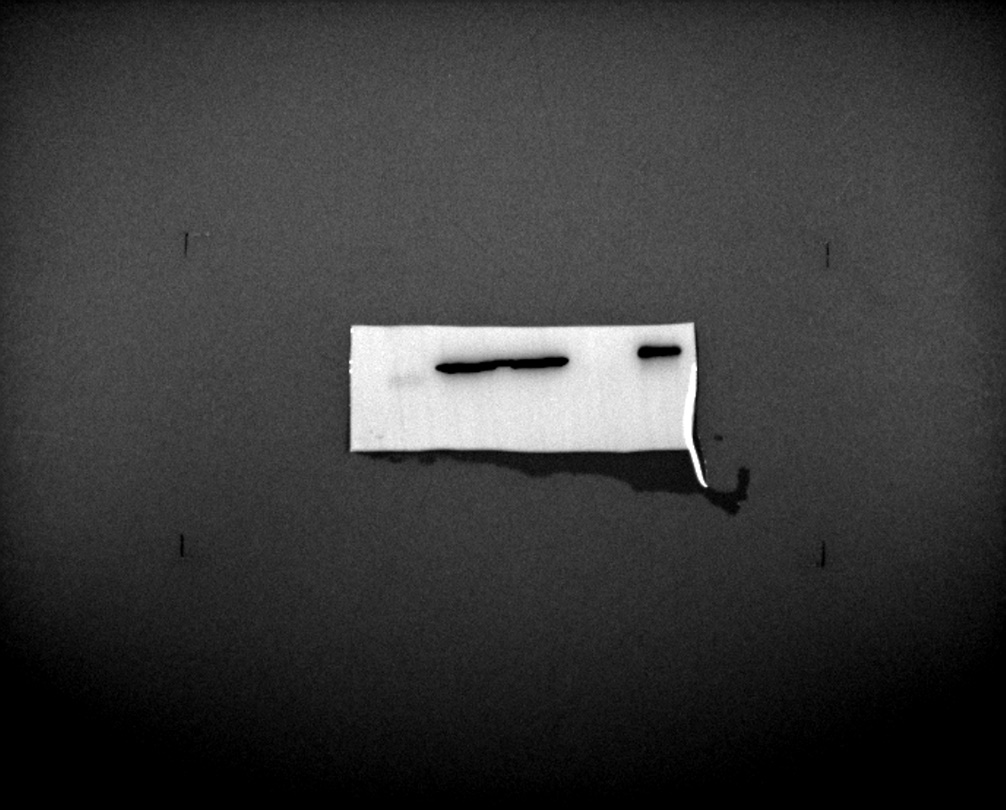

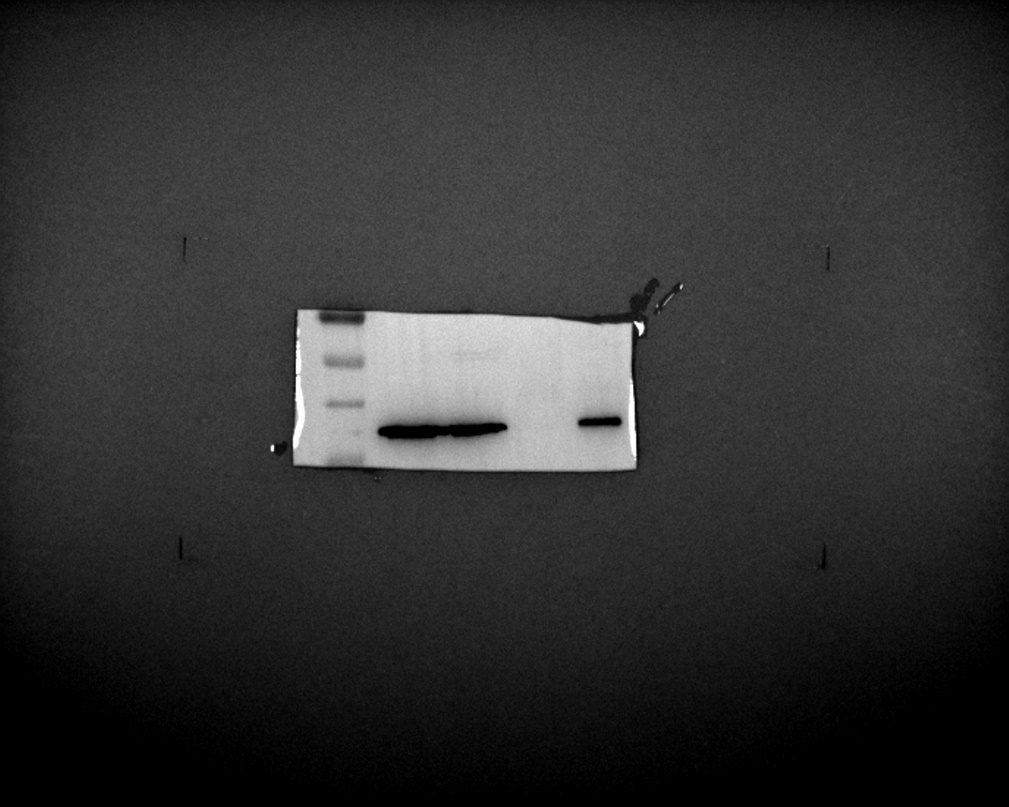

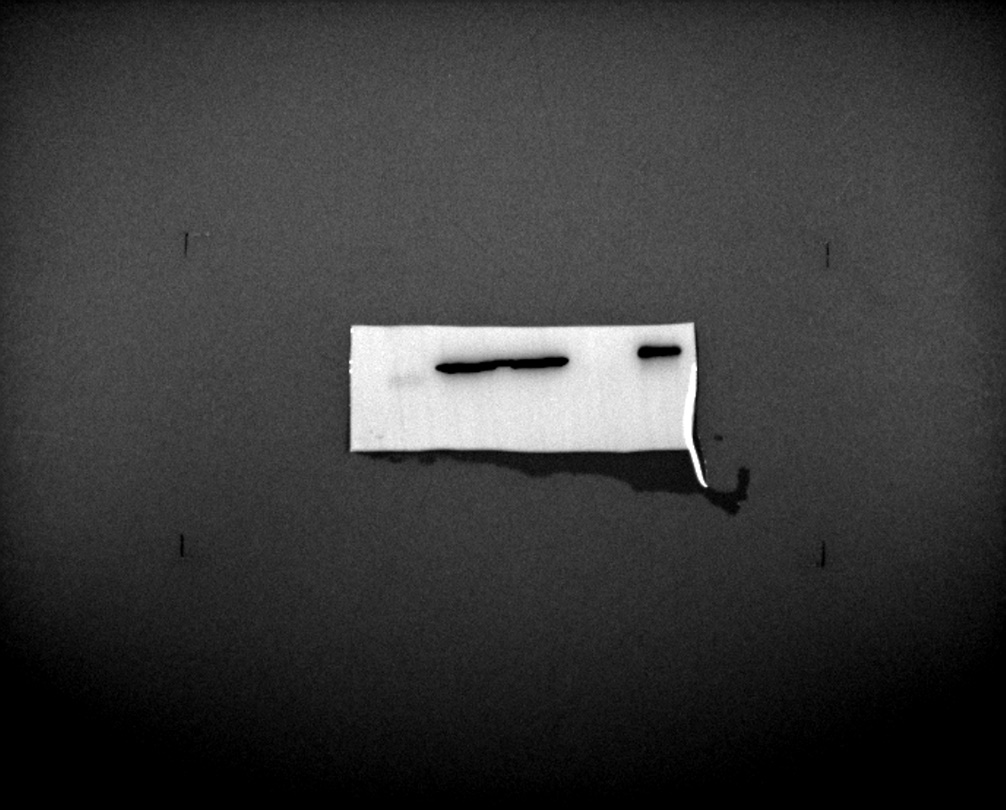

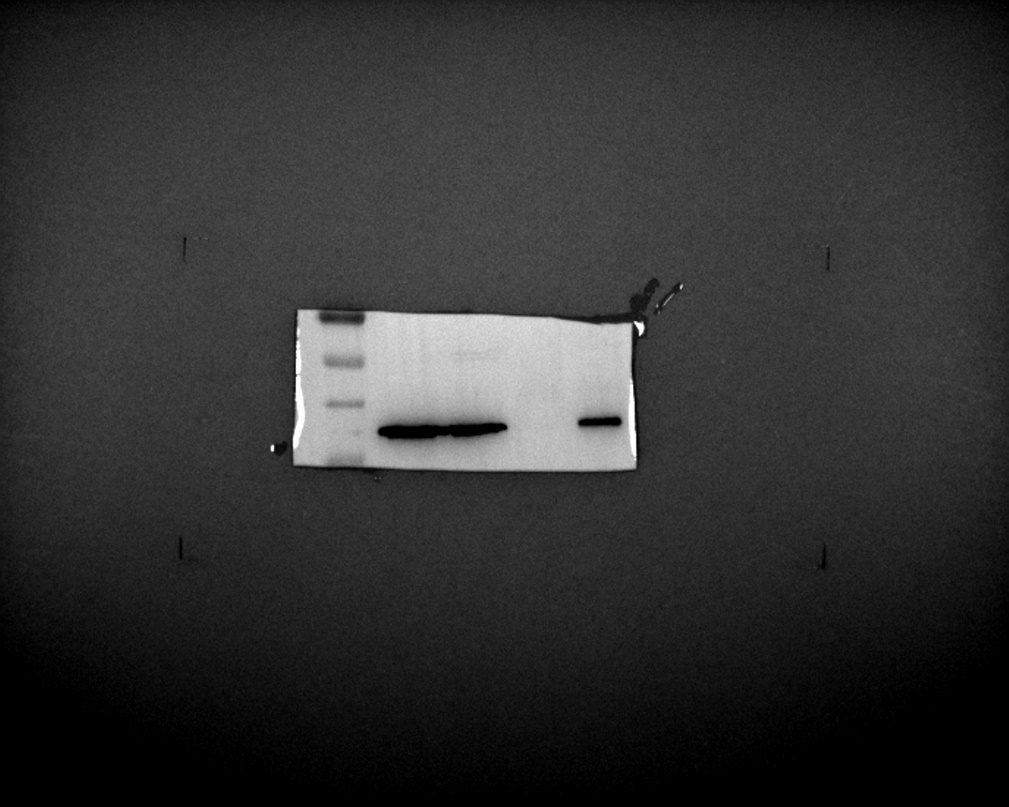


Two parts of the same gel

Two parts of the same gel

Myc-MAD2

Myc-MAD2

Flag-MAD2L1BP WT

Flag-MAD2L1BP MUT

25KD

35KD

70KD

55KD

42KD

35KD

Marker

Marker

35KD

25KD

35KD

42KD

55KD

70KD

Marker

Marker
