# Supplementary figures and images for: Biallelic variants in MAD2L1BP (p31comet) cause female infertility characterized by oocyte maturation arrest
Source: eLife. 2023 Jun 19;12:e85649. doi: 10.7554/eLife.85649 (PMC10319434; doi:10.7554/eLife.85649)

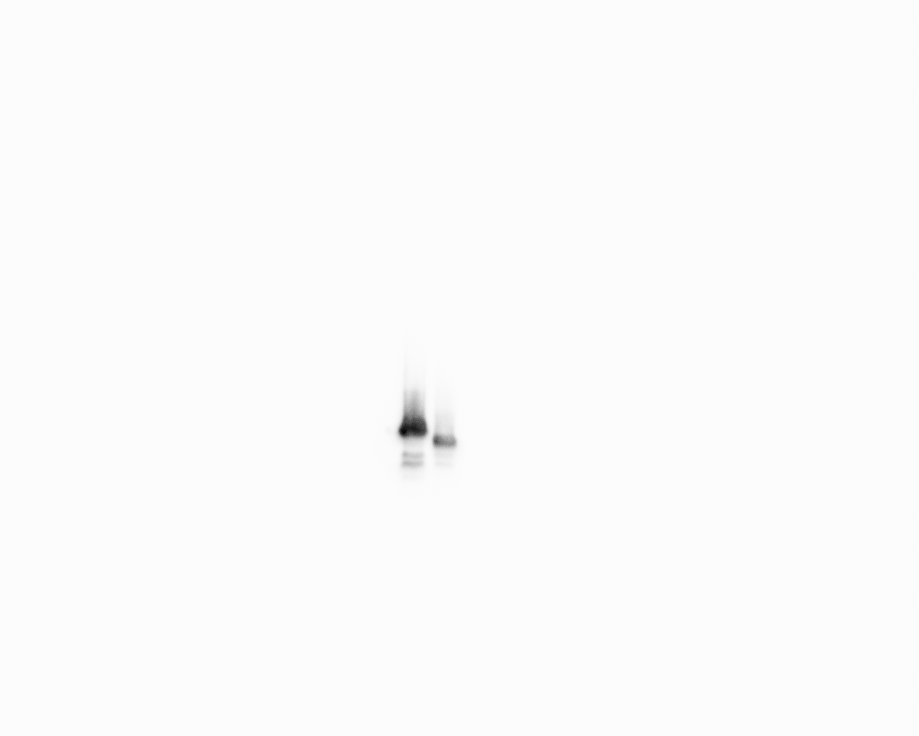

Supplement: Figure 2—source data 3. [file elife-85649-fig2-data3.zip › Figure 2-source data 3/Figure D-Anti-Flag.tif]

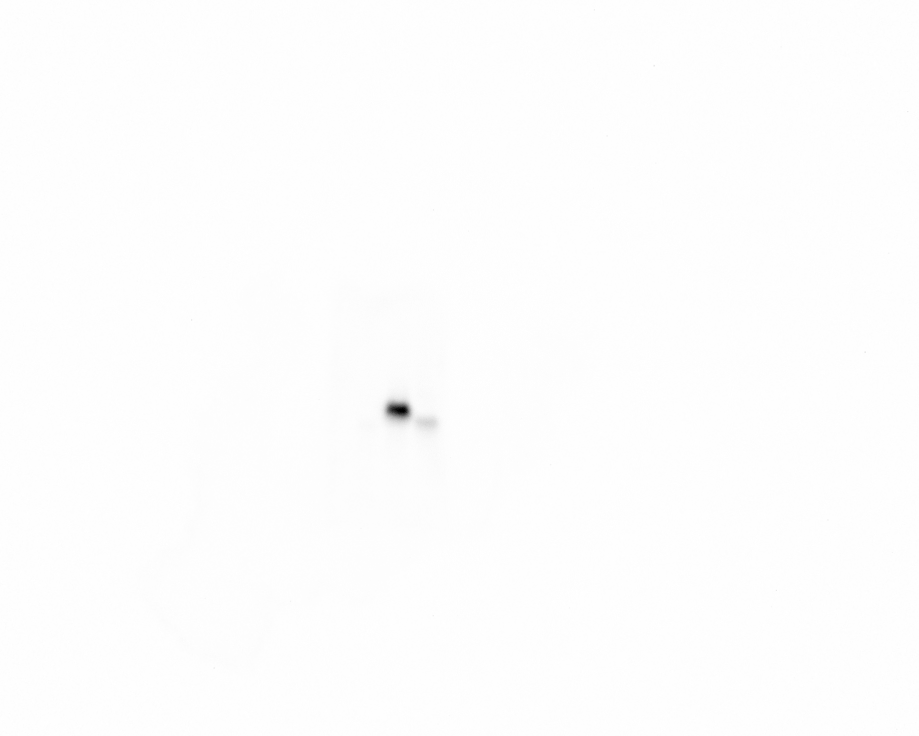

Supplement: Figure 2—source data 3. [file elife-85649-fig2-data3.zip › Figure 2-source data 3/Figure D-Anti-MAD2L1BP.tif]

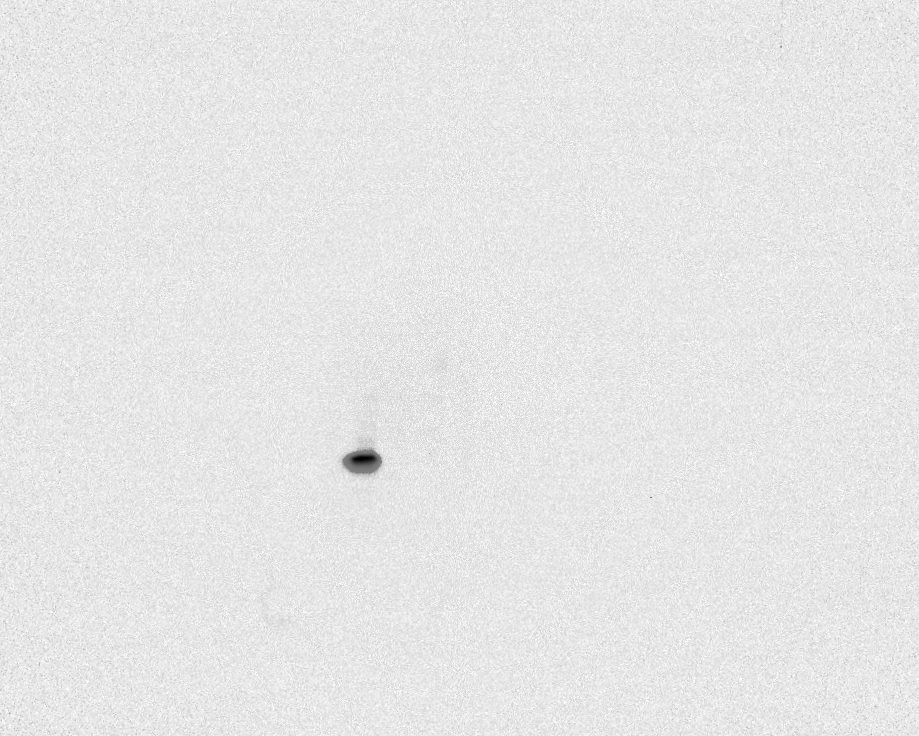

Supplement: Figure 2—source data 3. [file elife-85649-fig2-data3.zip › Figure 2-source data 3/Figure D-Anti-Myc.tif]

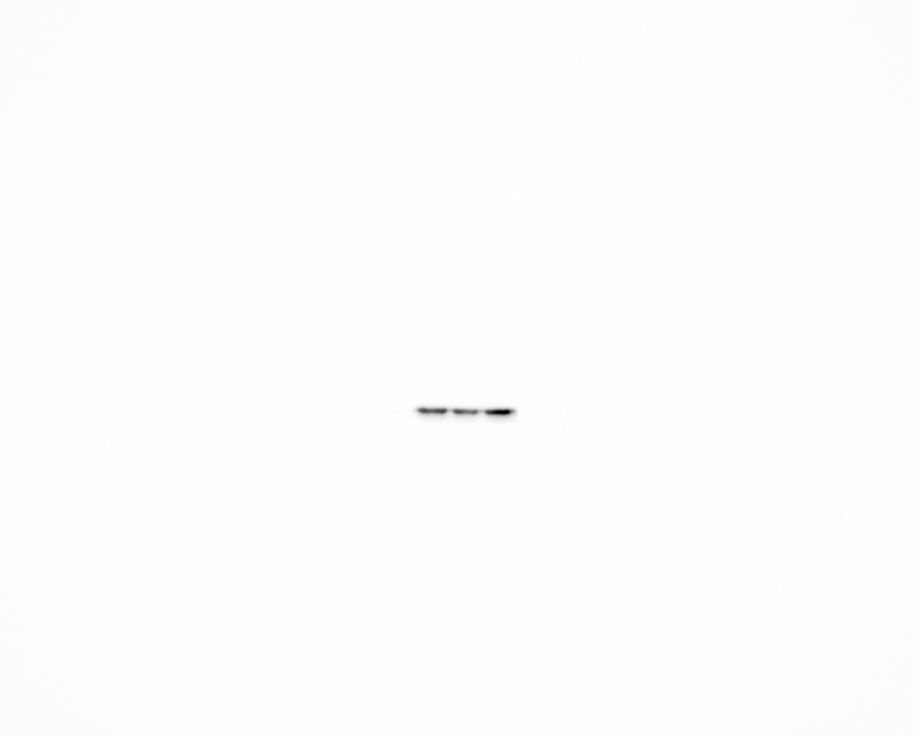

Supplement: Figure 2—source data 3. [file elife-85649-fig2-data3.zip › Figure 2-source data 3/Figure D-Anti-β-actin.tif]

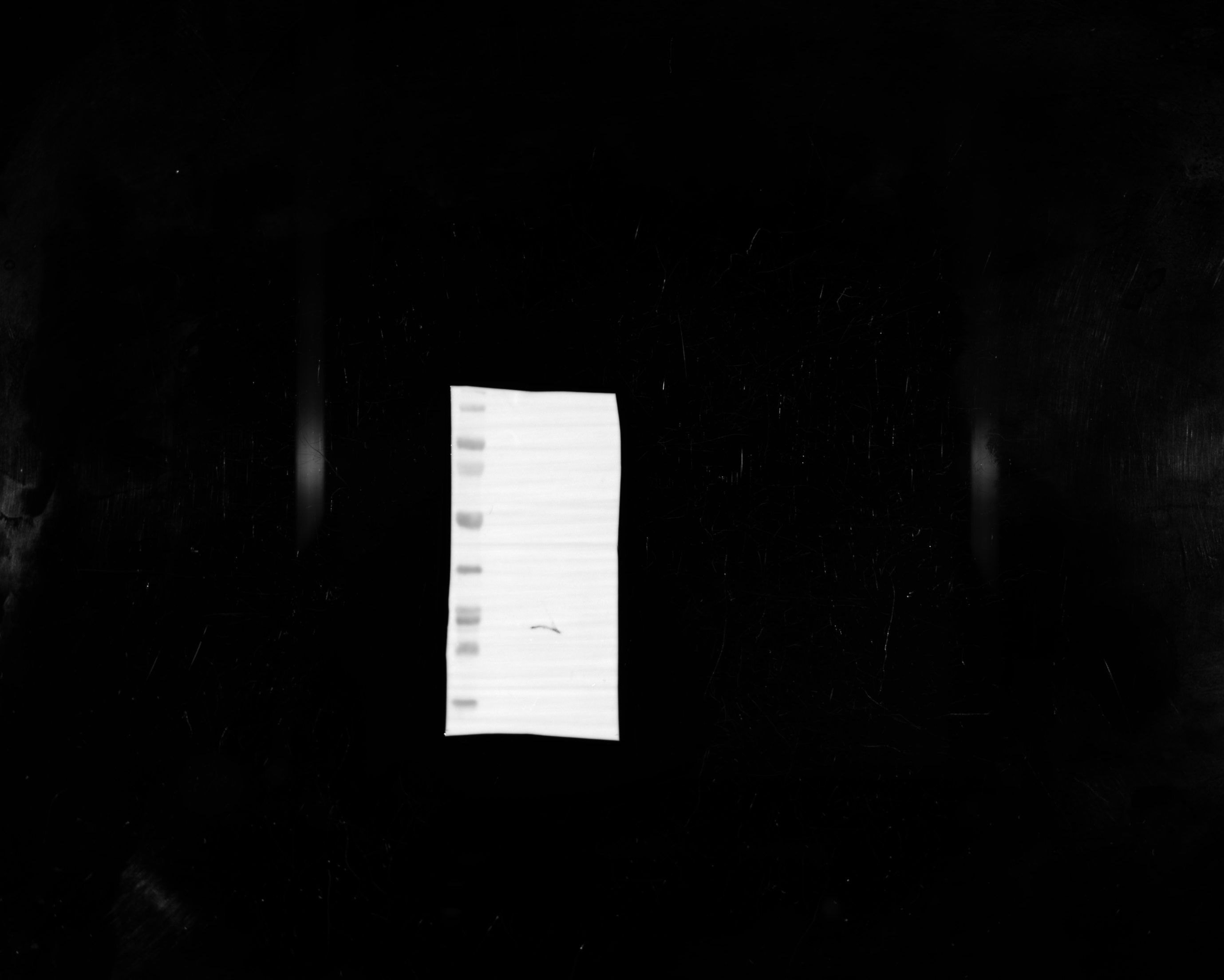

Supplement: Figure 2—source data 3. [file elife-85649-fig2-data3.zip › Figure 2-source data 3/Figure D-Marker-Anti-Flag.tif]

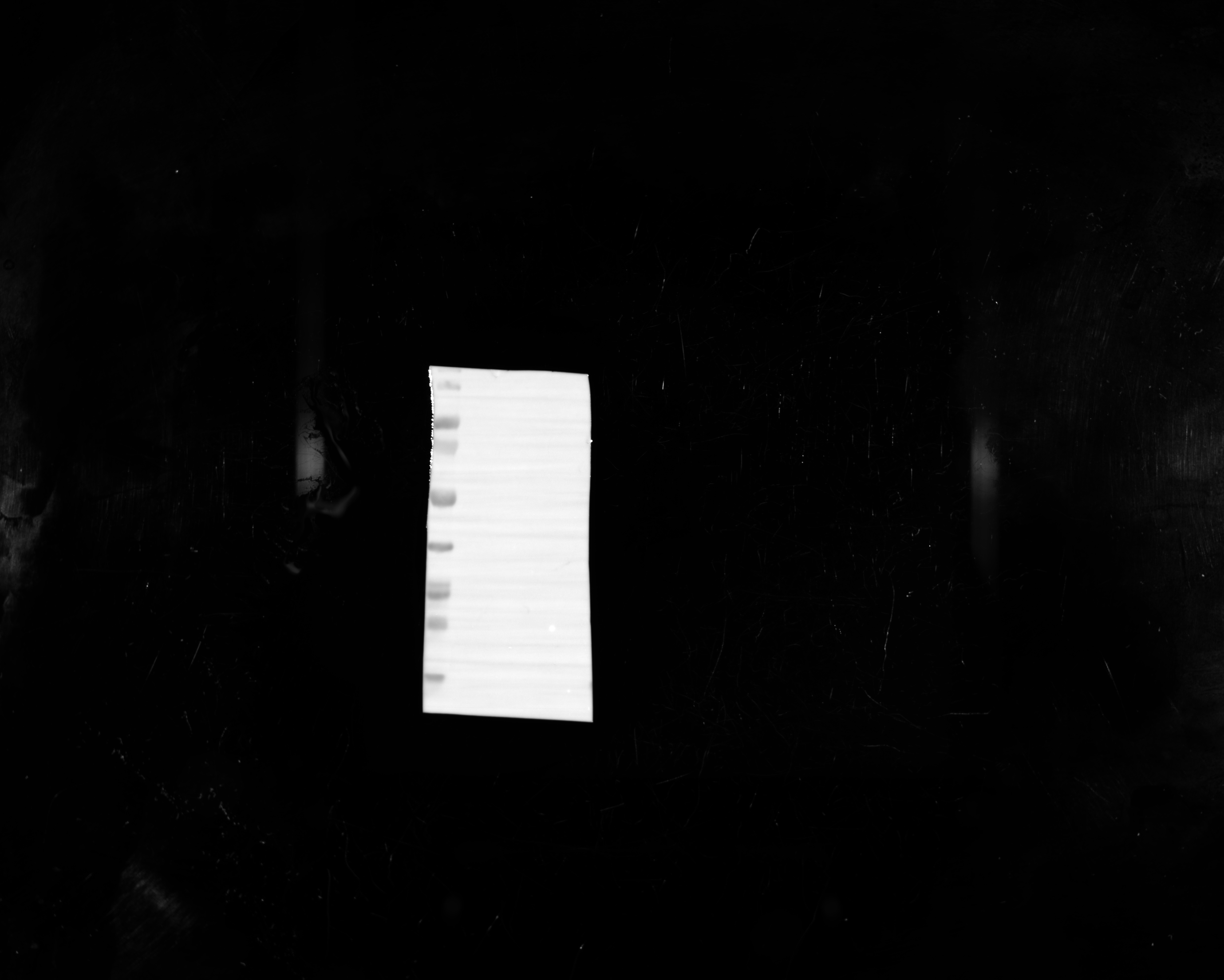

Supplement: Figure 2—source data 3. [file elife-85649-fig2-data3.zip › Figure 2-source data 3/Figure D-Marker-Anti-MAD2L1BP.tif]

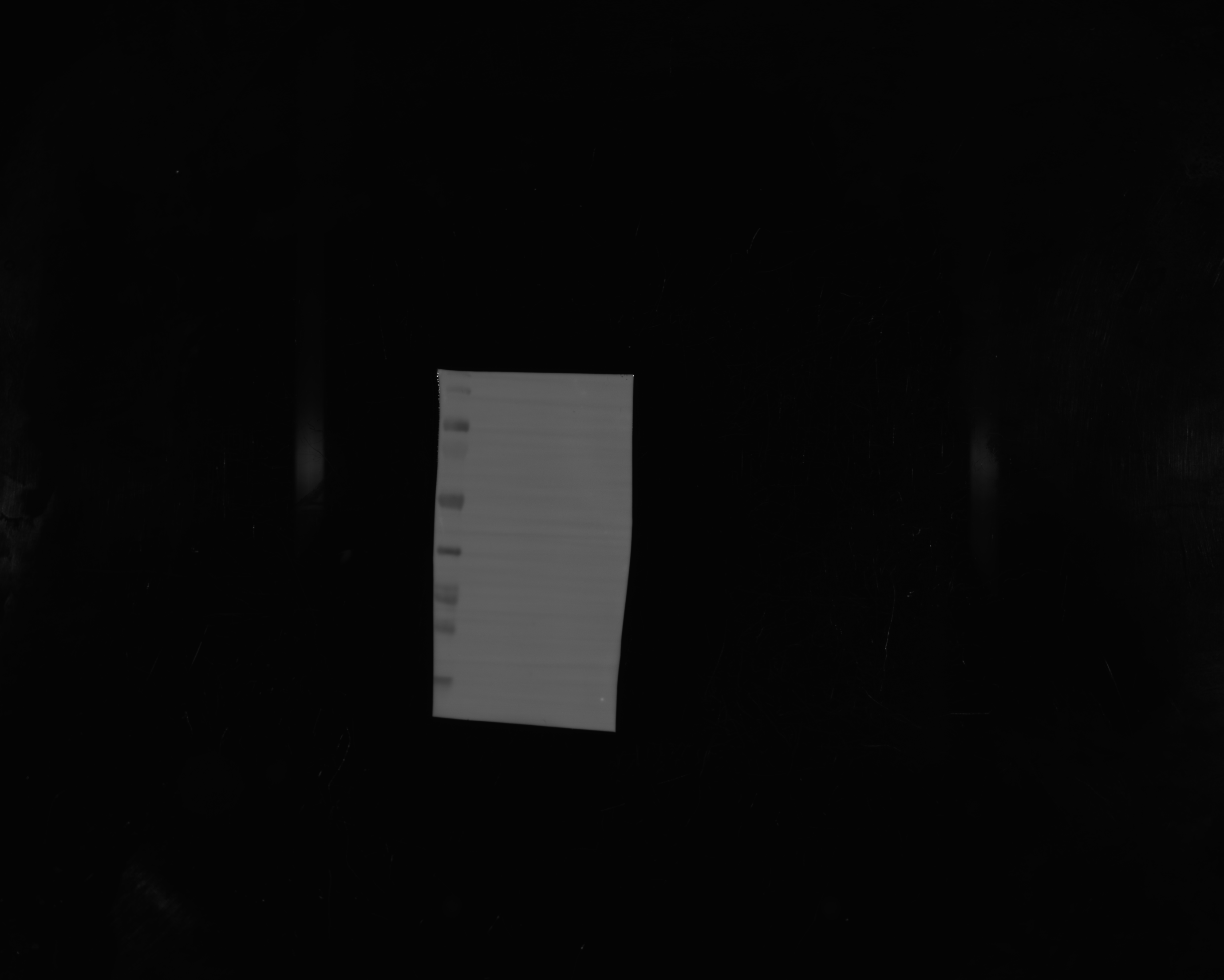

Supplement: Figure 2—source data 3. [file elife-85649-fig2-data3.zip › Figure 2-source data 3/Figure D-Marker-Anti-Myc.tif]

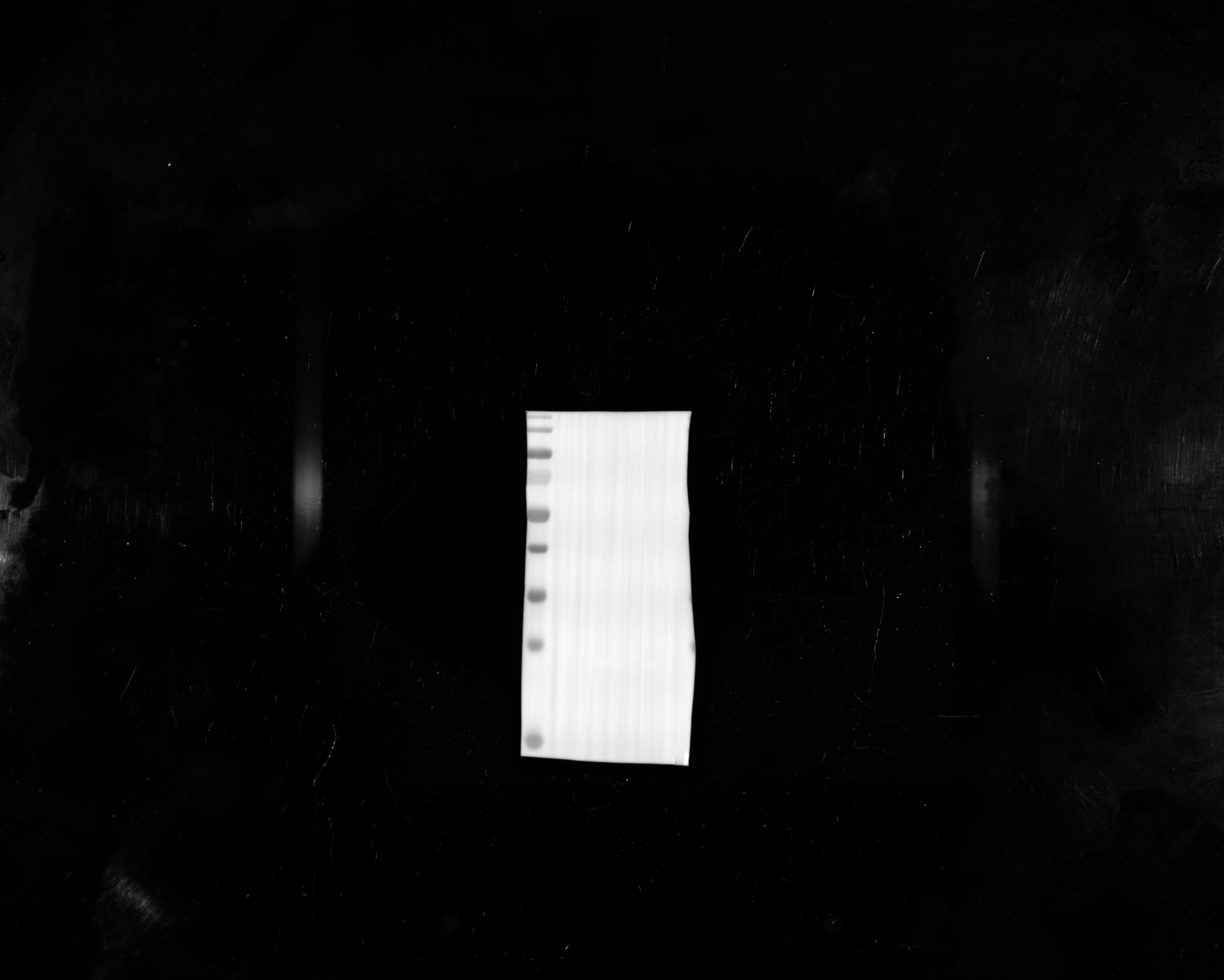

Supplement: Figure 2—source data 3. [file elife-85649-fig2-data3.zip › Figure 2-source data 3/Figure D-Marker-Anti-β-actin.tif]

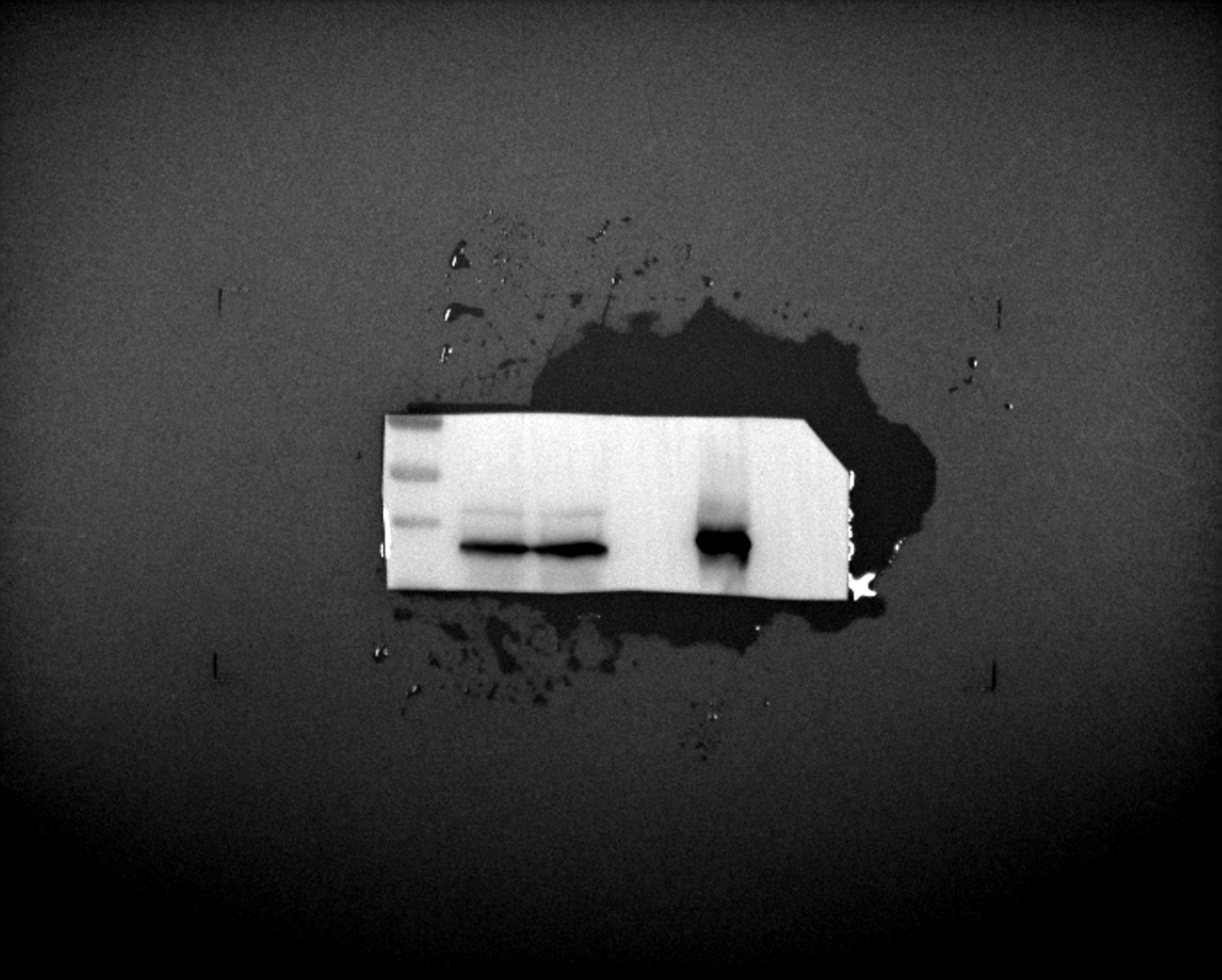

Supplement: Figure 2—source data 4. [file elife-85649-fig2-data4.zip › Figure 2-source data 4/Figure E-Flag-MAD2L1BP MUT.Tif]

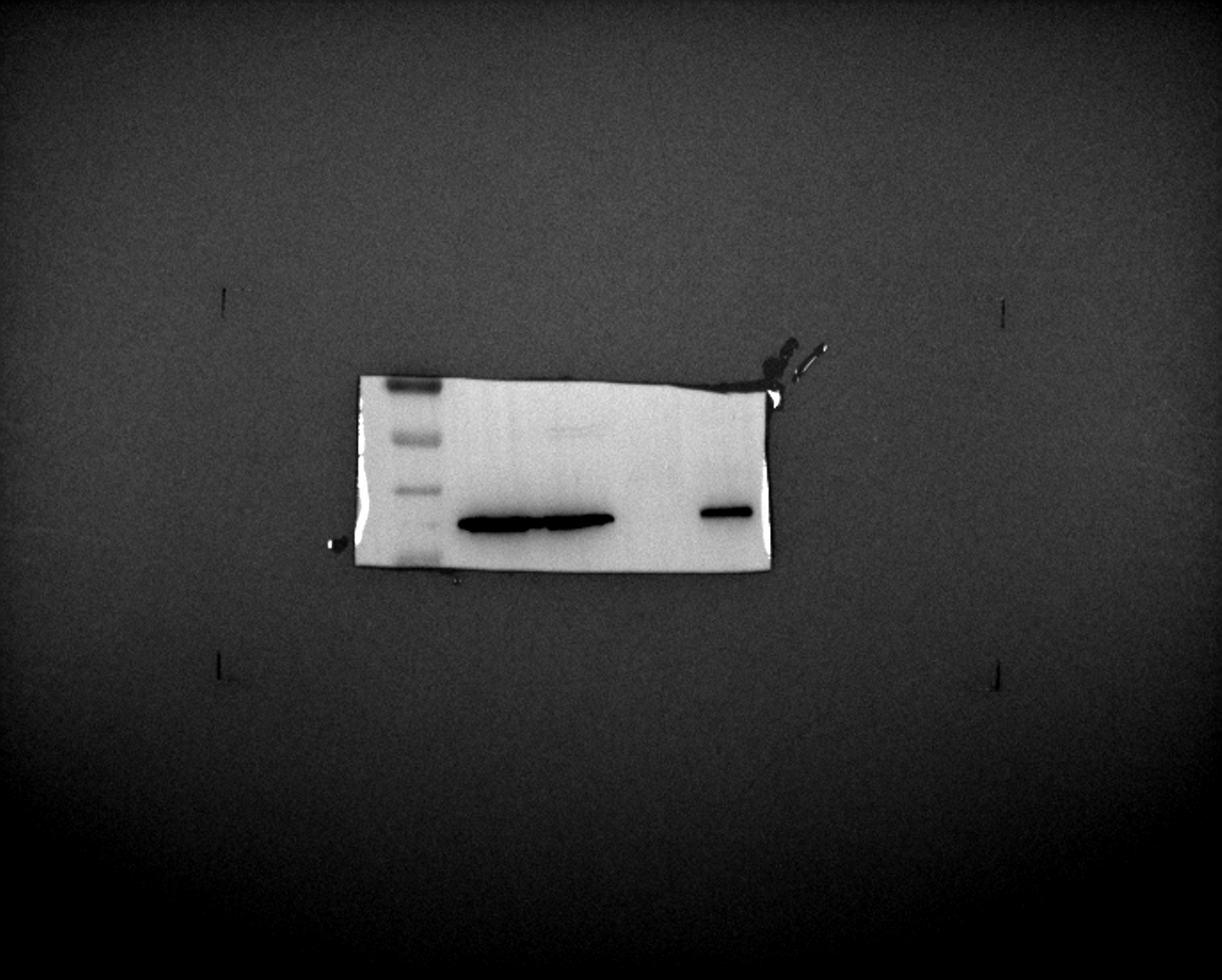

Supplement: Figure 2—source data 4. [file elife-85649-fig2-data4.zip › Figure 2-source data 4/Figure E-Flag-MAD2L1BP WT.Tif]

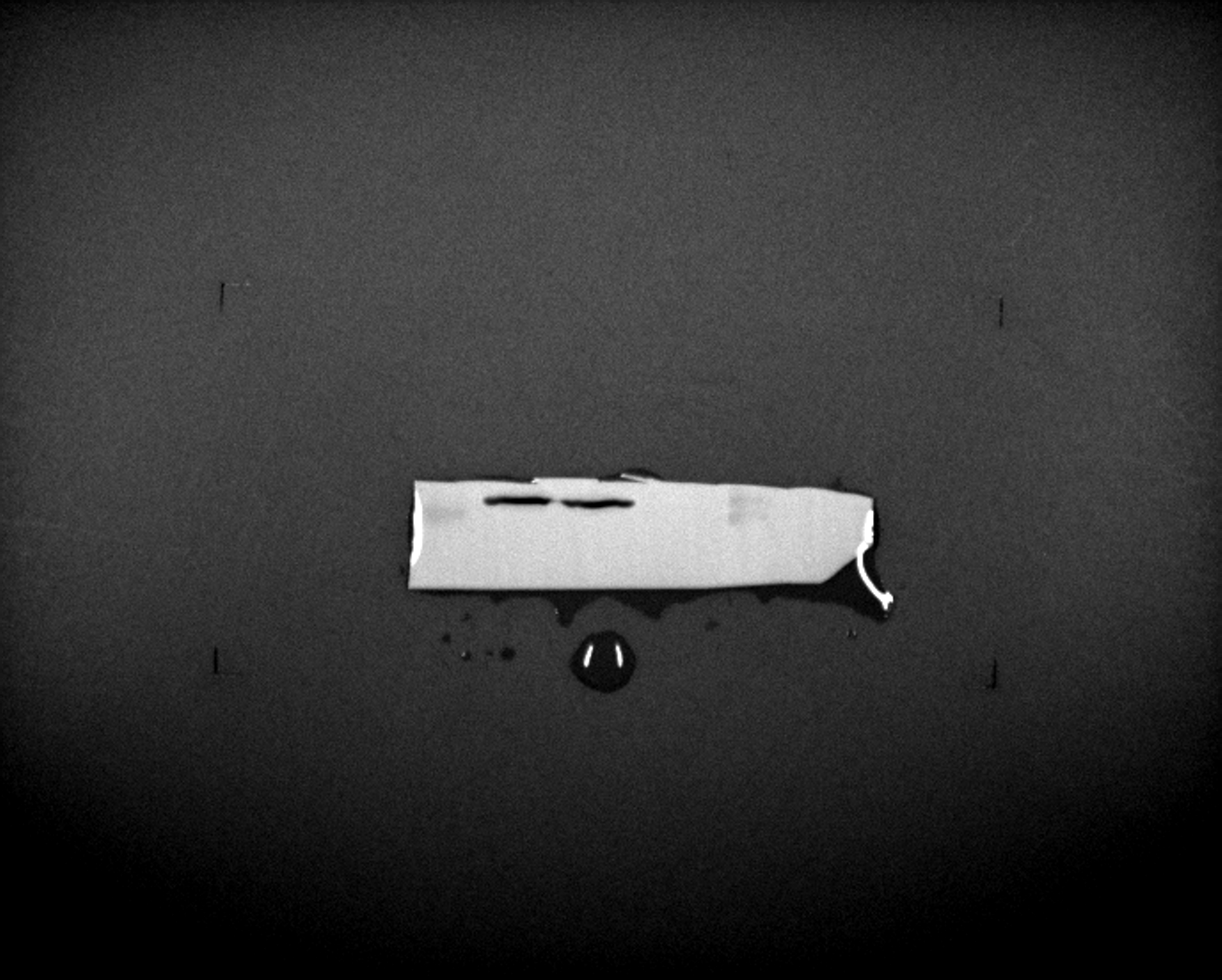

Supplement: Figure 2—source data 4. [file elife-85649-fig2-data4.zip › Figure 2-source data 4/Figure E-Myc-MAD2-bottom panel.Tif]

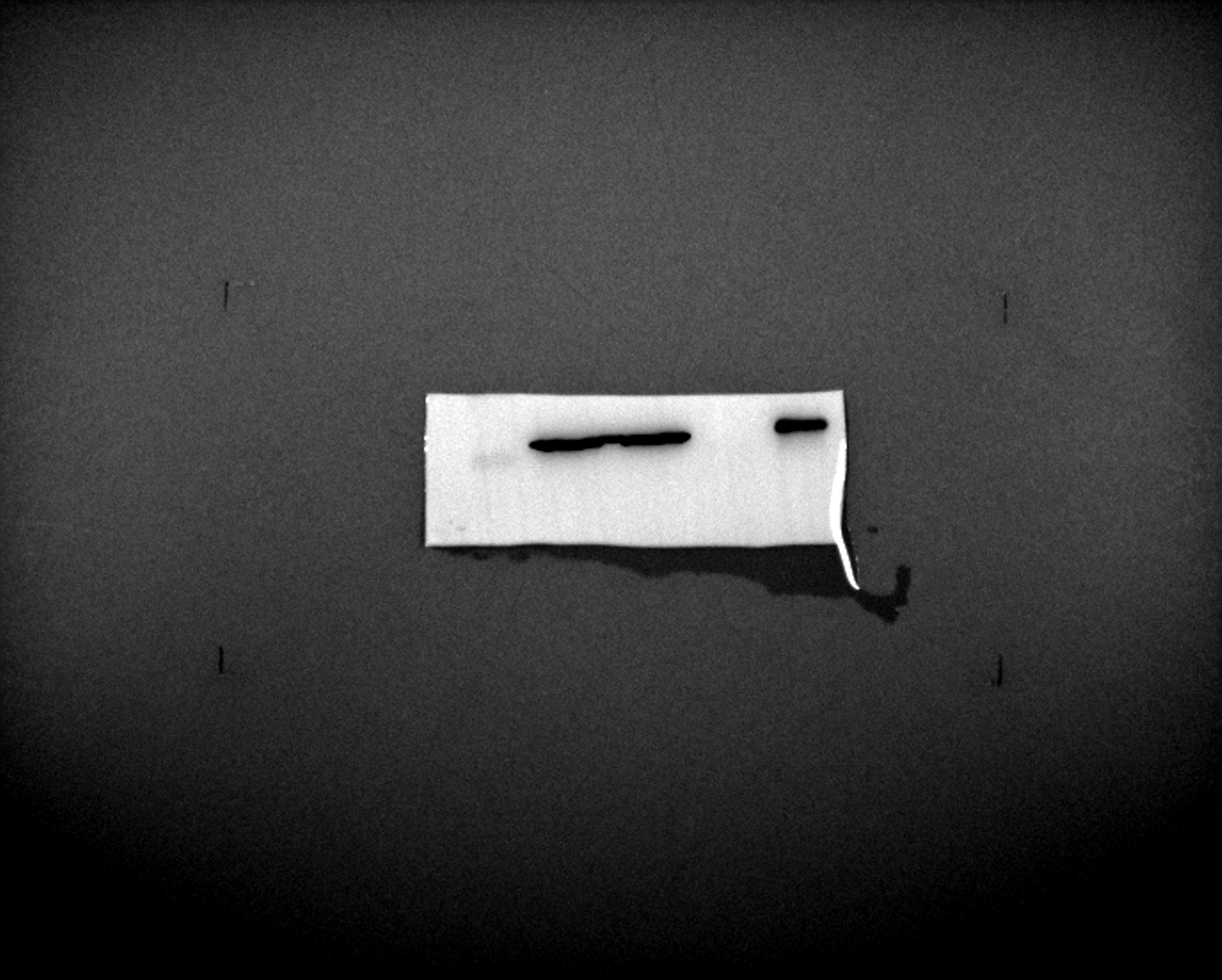

Supplement: Figure 2—source data 4. [file elife-85649-fig2-data4.zip › Figure 2-source data 4/Figure E-Myc-MAD2-top panel.Tif]
