# Supplementary material for: Biallelic variants in MAD2L1BP (p31comet) cause female infertility characterized by oocyte maturation arrest
Source: eLife. 2023 Jun 19;12:e85649. doi: 10.7554/eLife.85649 (PMC10319434; doi:10.7554/eLife.85649)
Supplement: Supplementary file 1. [file elife-85649-supp1.zip › Supplementary file/Supplementary file 1A.docx]

| Table S1. Semen parameters of the individual family 1(Ⅱ-2) with a homozygous mutation in *MAD2L1BP* | | |
| --- | --- | --- |
| Item | **Values** | **Normal value range^a^** |
| Semen volume (ml) | 1.0 | ≥ 1.4 |
| Sperm concentration (10^6^ per ml) | 0.8 | ≥ 15 |
| Total sperm number (10^6^ per ejaculate) | 0.8 | ≥ 39 |
| Total motility (PR+NP, %) | 11.1 | ≥ 40 |
| Progressive motility (PR, %) | 11.1 | ≥ 32 |
| Non-progressive motility (NP, %) | 0 |  |
| Immotile spermatozoa (IM, %) | 88.9 |  |
| a. Semen parameters were evaluated according to the World Health Organization 2010 guidelines. | | |
