# Supplementary material for: Biallelic variants in MAD2L1BP (p31comet) cause female infertility characterized by oocyte maturation arrest
Source: eLife. 2023 Jun 19;12:e85649. doi: 10.7554/eLife.85649 (PMC10319434; doi:10.7554/eLife.85649)
Supplement: Supplementary file 1. [file elife-85649-supp1.zip › Supplementary file/Supplementary file 1B.docx]

**Table S2. Genomic PCR primers used to amplify *MAD2L1BP* exons for Sanger sequencing**

| **Region** | **F/R** | **Primer Sequence (5’ to 3’)** | **PCR Size (bp)** |
| --- | --- | --- | --- |
| P1 + Exon 1 | F | CCGAGTACTGTGTGTGTGTCC | 1362 |
|  | R | GCCAGCCTGGTAATAAAGCA |  |
| Exon 1 + Int | F | TAGCGCGGATCCTAGACAAC | 713 |
|  | R | GCTGTGAATTGAGCCCTAGC |  |
| Exon 2 | F | GTTGCAGTGAGCCAAGATCA | 558 |
|  | R | AGGGACCAGAGGTCAAAACAC |  |
| P2 +Exon 1a | F | AACTCCTGGCCTCGGCAC | 1554 |
|  | R | AGCGGATAAACGAATGGATG |  |
| Exon 1a + Exon 3 | F | AACTGAGCCGGAAGTGGAG | 919 |
|  | R | CCTCCCATCTAAGAGCCACA |  |
| Exon 4 | F | CCCTGCCTTGTTCTTCTCTC | 720 |
|  | R | GCCATCAGCCTATCAGGAAA |  |

P1 represents the promoter region upward the Exon 1, Int represents the intronic region downward the Exon 1 and P2 represents the promoter upward the Exon 1a. F represents forward primers and R represents reverse primers.

**Table S3. Primers for quantitative PCR analysis of *MAD2L1BP***

| **Region** | **F/R** | **Primer Sequence (5’ to 3’)** | **PCR Size (bp)** |
| --- | --- | --- | --- |
| Exon 1 | F | TAGCGCGGATCCTAGACAAC | 243 |
|  | R | GCCAGCCTGGTAATAAAGCA |  |
| Exon 2 | F | TGCCATGCCCAGTCAGACC | 172 |
|  | R | AGCTACTTGGGAGGCTGAGG |  |
| Exon 1a | F | AACTGAGCCGGAAGTGGAG | 217 |
|  | R | AGCGGATAAACGAATGGATG |  |
| Exon 3 | F | CAGGAAGGCTGCTGTCAGTT | 176 |
|  | R | CCTCCCATCTAAGAGCCACA |  |
| Exon 4 | F | TCCTCCACCTTCAGAACACTG | 138 |
|  | R | TAGCTCAATGAACCGCCACA |  |

**Table S4. Primers for quantitative PCR analysis of human peripheral blood samples**

| **Gene** | **F/R** | **Primer Sequence (5’ to 3’)** | **PCR Size (bp)** |
| --- | --- | --- | --- |
| *MAD2L1BP* | F | AGCATATCATGTATCAACGCCAG | 153 |
|  | R | CCAGGGCTTGTTGGCATTTC |  |
| *MAD2* | F | GGACTCACCTTGCTTGTAACTAC | 249 |
|  | R | GATCACTGAACGGATTTCATCCT |  |
| *GAPDH* | F | TCGGAGTCAACGGATTTGGT | 181 |
|  | R | TTCCCGTTCTCAGCCTTGAC |  |

**Table S5. Primers for minigene assay**

| **Primer name** | **Primer Sequence (5’ to 3’)** |
| --- | --- |
| Splice RT F | AACAGGTTTTGGACCTCGAG |
| Splice RT R | ACTGGACTAGTGGATCCGAG |
